# Stable or improved neurological manifestations during miglustat therapy in patients from the international disease registry for Niemann-Pick disease type C: an observational cohort study

**DOI:** 10.1186/s13023-015-0284-z

**Published:** 2015-05-28

**Authors:** Marc C Patterson, Eugen Mengel, Marie T Vanier, Barbara Schwierin, Audrey Muller, Peter Cornelisse, Mercè Pineda, On behalf of the NPC Registry investigators

**Affiliations:** Department of Neurology, Mayo Clinic, 200 first Street SW, Rochester, MN 55905 USA; Villa Metabolica, University of Mainz, Mainz, Germany; INSERM Unit 820, Lyon, France; Actelion Pharmaceuticals Ltd, Allschwil, Switzerland; Fundació Hospital Sant Joan de Déu, CIBERER, Barcelona, Spain

**Keywords:** Niemann-Pick disease type C, Miglustat, Registry

## Abstract

**Background:**

Niemann-Pick disease type C (NP-C) is a rare neurovisceral disease characterised by progressive neurological degeneration, where the rate of neurological disease progression varies depending on age at neurological onset. We report longitudinal data on functional disease progression and safety observations in patients in the international NPC Registry who received continuous treatment with miglustat.

**Methods:**

The NPC Registry is a prospective observational cohort of NP-C patients. Enrolled patients who received ≥1 year of continuous miglustat therapy (for ≥90 % of the observation period, with no single treatment interruption >28 days) were included in this analysis. Disability was measured using a scale rating the four domains, ambulation, manipulation, language and swallowing from 0 (normal) to 1 (worst). Neurological disease progression was analysed in all patients based on: 1) annual progression rates between enrolment and last follow up, and; 2) categorical analysis with patients categorised as ‘improved/stable’ if ≥3/4 domain scores were lower/unchanged, and as ‘progressed’ if <3 scores were lower/unchanged between enrolment and last follow-up visit.

**Results:**

In total, 283 patients were enrolled from 28 centers in 13 European countries, Canada and Australia between September 2009 and October 2013; 92 patients received continuous miglustat therapy. The mean (SD) miglustat exposure during the observation period (enrolment to last follow-up) was 2.0 (0.7) years. Among 84 evaluable patients, 9 (11 %) had early-infantile (<2 years), 27 (32 %) had late-infantile (2 to <6 years), 30 (36 %) had juvenile (6 to <15 years) and 18 (21 %) had adolescent/adult (≥15 years) onset of neurological manifestations. The mean (95%CI) composite disability score among all patients was 0.37 (0.32,0.42) at enrolment and 0.44 (0.38,0.50) at last follow-up visit, and the mean annual progression rate was 0.038 (0.018,0.059). Progression of composite disability scores appeared highest among patients with neurological onset during infancy or childhood and lowest in those with adolescent/adult-onset. Overall, 59/86 evaluable patients (69 %) were categorized as improved/stable and the proportion of improved/stable patients increased with age at neurological onset. Safety findings were consistent with previous data.

**Conclusions:**

Disability status was improved/stable in the majority of patients who received continuous miglustat therapy for an average period of 2 years.

## Background

Niemann-Pick disease type C (NP-C) is a rare neurovisceral disease characterized by progressive neurodegeneration [1, 2]. NP-C is associated with a highly heterogeneous spectrum of neurological manifestations that can start at any age [2, 3]. Most published evidence on the disease relates to patients with disease onset during infancy and childhood, but epidemiological data accumulated over the last two decades has revealed an increase in the number of adult-onset cases diagnosed [1, 2]. Disease progression has been shown to depend on age at onset of neurological manifestations, with early-onset forms progressing faster than later onset forms [1].

Miglustat (Zavesca®, Actelion Pharmaceuticals Ltd) is a glucosylceramide synthase inhibitor that was initially approved for the treatment of Gaucher disease type I [4]. Based on data from a randomized controlled clinical trial [5], long-term extension studies [6, 7], and a retrospective observational cohort study [8], miglustat was approved for the treatment of progressive neurological manifestations in pediatric and adult patients with NP-C in the EU in 2009 [4], and has since been approved in a number of other countries. Observational cohort studies, case series, and case reports have since supported trial findings [9–13].

Here we report longitudinal data on functional disease progression and safety observations from patients who have been observed in the NPC Registry for ≥1 year and who were treated continuously with miglustat during that period.

## Methods

The international NPC Registry was initiated in May 2009 as a post-approval commitment to the European Medicines Agency (EMA) following approval of a new indication for miglustat for the treatment of progressive neurological deterioration in adults and children with NP-C [4]. The Registry is a prospective, observational cohort study conducted in clinical practice settings. All patients with a diagnosis of NP-C are eligible for enrolment, regardless of the treatment they receive. A previous published report from this Registry described general methodological details and patient characteristics at enrolment [14].

The NPC Registry collects data on demographics, diagnosis, disease characteristics and treatment. Before entering any clinical visit data, written informed consent is obtained from all patients (including children) and/or their legal guardians.

For the current longitudinal study, data were analyzed for patients that: 1) received miglustat therapy continuously between enrolment visit and last follow-up visit (where ‘continuously’ was defined as at least 90 % of the observation period with no single period without miglustat lasting more than 28 days); and 2) had a minimum of 1 year of observation time in the Registry.

Functional disability status is recorded at enrolment and at each follow-up visit in the Registry using an expert-based disease-specific disability scale that assesses four key neurological domains in NP-C (ambulation, manipulation, language and swallowing) [15]. The scoring system of this scale originally ranged from 0 (normal) to 4 or 5 (worst) [16], but has since been modified to provide an equal weighting in each domain, and ranges from 0 (normal) to 1 (worst) [8, 15]. The 0–1 scoring system was chosen for use in the Registry [14] as this scale has been employed in key studies on the natural history of NP-C [8, 15], is listed among the useful assessment tools in the international recommendations for managing NP-C [2, 3], and has been employed in describing miglustat treatment outcome on NP-C disease progression in previous national cohorts [12].

For the current analysis, composite disability scores were calculated as the mean of all four domains. Disease progression was assessed by two statistical methods: 1) annual progression rates were calculated as the change in composite disability score from enrolment to last follow-up visit divided by time in years from enrolment to last follow-up visit; 2) categorical analysis, where patients were categorized as ‘improved/stable’ when at least three out of four individual disability domain scores were decreased or unchanged between enrolment and last follow-up visit, and patients with fewer than three out of four domain scores decreased or unchanged were categorized as ‘progressing’ [8, 15].

Statistical analyses are descriptive. All percentages were calculated relative to the number of patients with available data per parameter. Data are presented for all patients and stratified according to internationally agreed age at neurological onset categories (early-infantile onset [<2 years], late-infantile onset [2 – <6 years], juvenile onset [6 – <15 years], and adolescent/adult onset [≥15 years]) [1, 2]. One modification to these internationally agreed neurological onset categories was that in this analysis all patients with onset aged <2 years were included in the early-infantile subgroup due to the low number of cases. The participation rate of miglustat-treated patients in Europe was calculated as the number of miglustat-treated patients enrolled in the Registry divided by the estimated number of NP-C patients treated with miglustat in European countries.

## Results

### Patients

As of the data extraction on 19 October 2013, a total of 283 patients from 28 centers in 13 European countries, Canada and Australia were enrolled in the NP-C Registry. The majority of patients were enrolled in Germany, the UK and France.

**Fig. 1:**
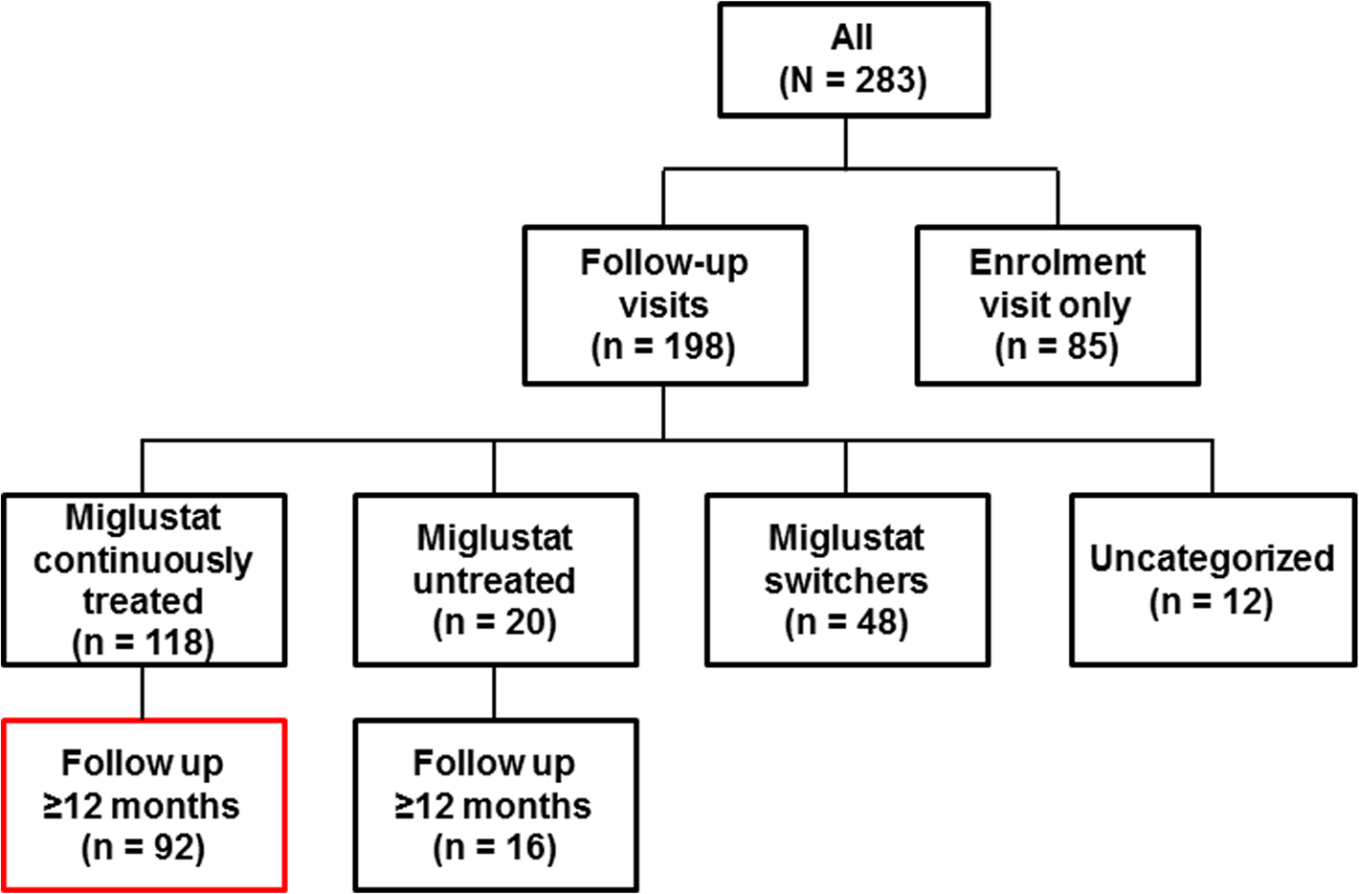
NPC Registry patient summary. Red box indicates patient cohort analyzed in this paper

Ninety-two patients received continuous miglustat therapy between enrolment and last follow-up visit, and were observed for at least 1 year (Fig. 1). The mean (SD) observation period was 2.0 (0.7) years. From this point onwards, all data refer to this continuously treated patient group (N = 92).

Patient characteristics at enrollment stratified by age at neurological onset categories are summarized in Table 1. Amongst all patients, the mean (SD) age at neurological onset was 9.8 (8.6) years, ranging from <1 year to 44.6 years, and the mean (SD) age at diagnosis was 13.7 (10.9) years, ranging from 0.1–44.7 years. Two patients in the late-infantile subgroup were diagnosed after 20 years of age; initial neurological symptoms observed between 2 and 6 years of age were ‘extended daytime sleep periods’ and clumsiness in one patient, and ’coordination disturbances’ in the other patient.

**Table 1:** Demographics and disease characteristics of all patients receiving continuous miglustat therapy for at least 12 months in the Registry by neurological onset category

	Patients continuously treated with miglustat (N = 92)	Patients with age at neurological onset data (n = 84)			
		EI (<2 years)	LI (2 – <6 years)	JUV (6 – <15 years)	AA (≥15 years)
Male: female, n (%)	47 (51.1): 45 (48.9)	5 (55.6): 4 (44.4)	12 (44.4): 15 (55.6)	14 (46.7): 16 (53.3)	10 (55.6): 8 (44.4)
*Age at onset of neurological manifestations*					
n	84*	9	27	30	18
Mean (SD), years	9.8 (8.6)	0.9 (0.7)	4.2 (1.3)	9.8 (2.7)	22.7 (8.8)
Median (range), years	6.9 (<1–44.6)	1.0 (<1 – 2.0)	4.5 (2.0 – 5.9)	9.2 (6.1 – 14.6)	18.7 (15.5 – 44.6)
*Age at diagnosis*					
n	87^‡^	9	27	28^#^	17^†^
Mean (SD), years	13.7 (10.9)	1.9 (1.9)	7.8 (7.0)	16.6 (7.7)	26.7 (9.9)
Median (range), years	12.0 (0.1–44.7)	1.4 (0.1 – 5.9)	5.2 (0.1 – 28.2)**	13.7 (7.7 – 40.2)	22.1 (14.4 – 44.7)
*Age at enrolment*					
n	92	9	27	30	18
Mean (SD), years	18.9 (11.3)	4.7 (2.3)	12.5 (7.2)	20.7 (8.3)	31.2 (9.2)
Median (range), years	18.8 (0.7–48.3)	4.1 (2.2–8.6)	11.1 (3.6–29.4)	20.7 (9.2–41.5)	29.0 (18.7–48.3)
*Composite disability score at enrolment*					
n	87^††^	9	26^α^	29^α^	18
Mean (SD)	0.37 (0.23)	0.43 (0.33)	0.32 (0.24)	0.45 (0.22)	0.35 (0.17)
Median (range)	0.35 (0–1.00)	0.52 (0 – 1.00)	0.28 (0 – 1.00)	0.44 (0 – 0.88)	0.29 (0.06 – 0.81)
*Composite disability score at last follow up*					
n	90^β^	9	27	30	17
Mean (SD)	0.44 (0.27)	0.59 (0.36)	0.46 (0.29)	0.49 (0.24)	0.31 (0.22)
Median (range)	0.36 (0–1.00)	0.58 (0.06–1.00)	0.38 (0.06–1.00)	0.51 (0.00–0.94)	0.29 (0.00–0.88)

*Eight patients had no age at neurological onset data (two with no neurological manifestations at enrolment and six with unknown neurological status); ^‡^five patients had no age at diagnosis data, including three for whom age at neurological onset was not available; ^#^two patients had no age at diagnosis data; ^†^one patient had no age at diagnosis data; **two patients were diagnosed at age >20 years (one had extended daytime sleep periods and clumsiness and the other had coordination disturbances reported at time of first neurological symptoms). ^††^five patients had insufficient data, including two for whom age at neurological onset was not available. ^α^one patient had insufficient data on composite disability score at enrolment; ^β^two patients had insufficient disability score data at follow up. Note: patient age at diagnosis is calculated based on date of the first positive filipin staining or gene mutation analysis result. EI, early-infantile onset; LI, late-infantile onset; JUV; juvenile onset; AA, adolescent/adult onset

Overall the mean (SD) composite disability score at enrolment was 0.37 (0.23), with scores ranging over the full scale from 0 (normal function) to 1.0 (maximum impairment) (Table 1).

### Neurological and visceral manifestations prior to or at enrolment

Almost all patients with available medical history data had one or more neurological manifestation. The distribution of the most common manifestations was as follows: ataxia (70 %); vertical gaze palsy (68 %); dysarthria (63 %); cognitive impairment (56 %); dysphagia (44 %); dystonia (43 %); seizures (33 %); and cataplexy (24 %). As regards visceral symptoms, a history of neonatal jaundice was reported in 58 % of patients, hepatomegaly during infancy was reported in 35 %; and splenomegaly during infancy was reported in 51 %.

### Treatment

Amongst the 92 miglustat-treated patients, 82 (89 %) received miglustat therapy prior to enrolment, nine (10 %) initiated miglustat therapy at enrolment, and one patient started miglustat shortly after enrolment. The mean (SD) total miglustat exposure between miglustat start and last follow-up visit was 3.9 (1.9) years (Table 2), where ‘miglustat start’ was defined as the start of the most recent, uninterrupted miglustat therapy period prior to enrolment. Mean (SD) miglustat exposure prior to enrolment was 1.9 (1.8) years (range 0–7.6 years). Mean (SD) miglustat exposure during the observation period (i.e., from enrolment to last follow-up visit) was 2.0 (0.7) years. Mean treatment exposure during the observation period and mean exposure prior to enrolment were similar across the neurological-onset subgroups.

**Table 2:** Miglustat exposure among continuously treated patients by neurological onset category

	All patients continuously treated with miglustat (N = 92)	Patients with age at neurological onset and treatment data			
		EI (<2 years)	LI (2 – <6 years)	JUV (6 – <15 years)	AA (≥15 years)
Age at 1st miglustat dose, years					
n*	82	9	23	27	15
Mean (SD)	16.1 (10.8)	2.9 (1.9)	9.1 (6.9)	19.6 (8.2)	27.1 (8.0)
Range	0.7–44.6	0.8 − 7.4	2.1 − 28.5	9.8 − 41.2	16.3 − 44.6
Pre-enrolment miglustat exposure**, years					
n*	91	9	27	29	18
Mean (SD)	1.9 (1.8)	1.7 (1.5)	2.4 (2.1)	1.5 (1.5)	1.7 (1.9)
Range	0–7.6	0.3 − 4.5	0 − 7.6	0 − 4.6	0 − 6.0
*Miglustat exposure during follow up* ^*†*^ *, years*					
n*	92	9	27	30	18
Mean (SD)	2.0 (0.7)	1.8 (0.4)	1.9 (0.6)	2.3 (0.7)	1.9 (0.5)
Range	1.0–3.7	1.4–2.8	1.1–3.3	1.0–3.7	1.0–3.2
*Overall miglustat exposure* ^*‡*^ *, years*					
n*	91	9	27	29	18
Mean (SD)	3.9 (1.9)	3.4 (1.42)	4.3 (2.2)	3.9 (1.7)	3.6 (1.9)
Range	1.1–9.8	2.1 − 6.0	1.6 − 9.8	1.3 − 6.8	1.3 − 7.9

*Number of patients with available data (eight patients who had no age at neurological onset data are not included in the age-at-onset subgroup columns); **exposure period from most recent miglustat treatment start date up to and including NPC Registry enrolment day; ^†^period from Registry start to last follow-up visit; ^‡^period from most recent miglustat treatment start date prior to enrolment up to last follow-up visit in the NPC Registry. EI, early-infantile onset; LI, late-infantile onset; JUV; juvenile onset; AA, adolescent/adult onset

### Disease progression

**Fig. 2:**
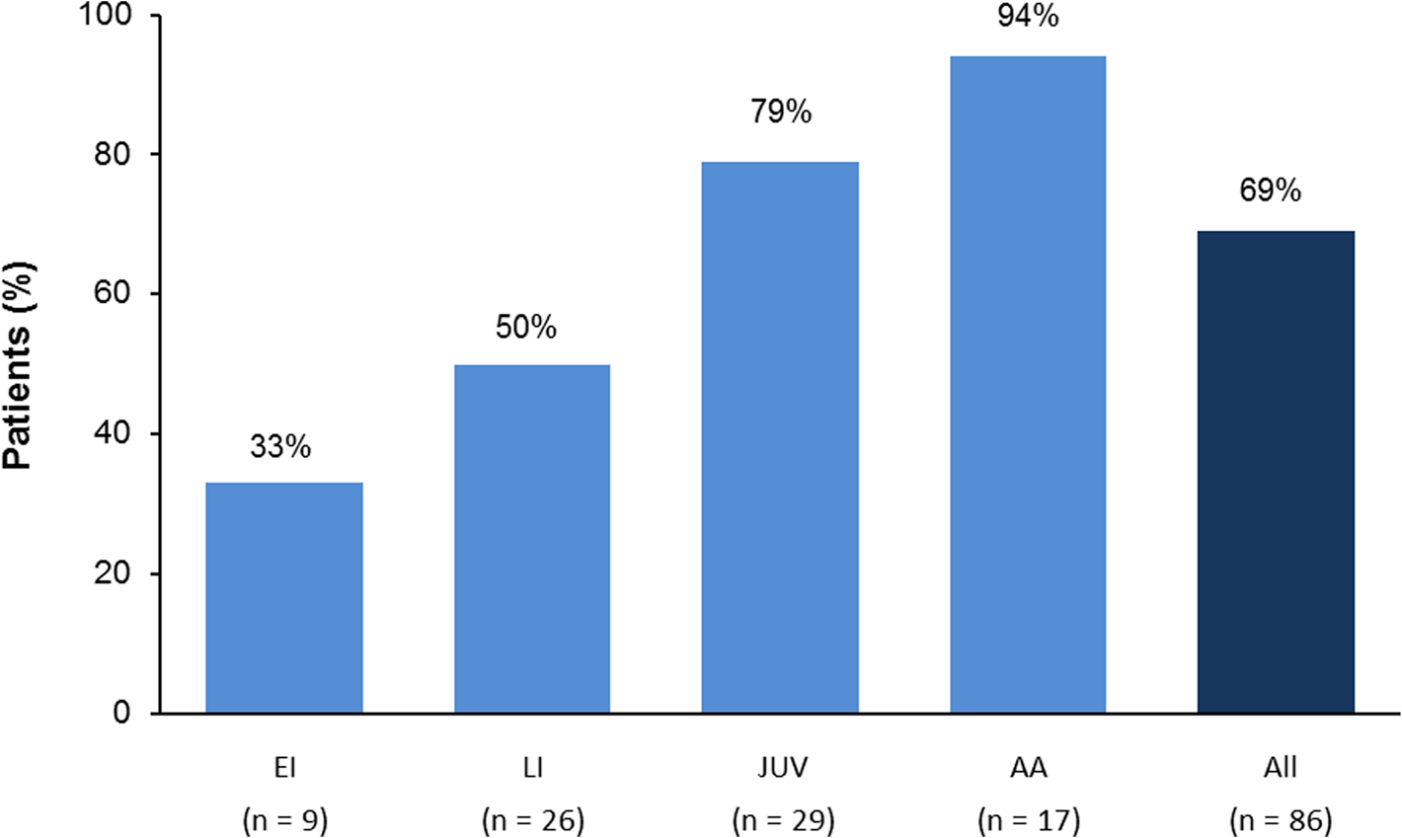
Proportions of patients who received continuous miglustat therapy during follow up* and were categorized as ‘improved/stable’. *The length of the observation period varied for each patient; data values based on all patients with available data, per age at neurological onset subgroup. EI, early-infantile onset; LI, late-infantile onset; JUV; juvenile onset; AA, adolescent/adult onset. Note: six patients had insufficient disability scale data

Eighty-six patients had available data for the categorical analysis of disease progression by age at neurological onset. Ambulation, manipulation, language and swallowing were improved (i.e., decreased score) or stable (i.e., no change in score) in 75 %, 71 %, 77 % and 74 % of patients, respectively. Overall, two-thirds (n = 59; 69 %) were categorized as improved/stable (as defined by at least three out of four disability domains being decreased or unchanged between enrolment and last follow-up visit). The proportion of improved/stable patients increased across neurological onset categories: early-infantile (33 %) < late-infantile (50 %) < juvenile (79 %) < adolescent/adult onset (94 %) (Fig. 2).

**Fig. 3:**
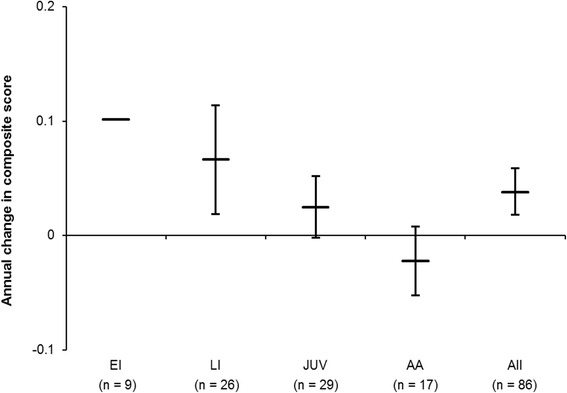
Annual progression rate in patients who received continuous miglustat therapy between enrolment and last follow up. Data are means ± 95 % CI; no 95 % CI could be calculated for the early infantile-onset group due to the low number of patients. EI, early-infantile onset; LI, late-infantile onset; JUV; juvenile onset; AA, adolescent/adult onset. Note: six patients had insufficient disability scale data.

The mean (95 % CI) composite disability score among all patients was 0.37 (0.32, 0.42) at enrolment and 0.44 (0.38, 0.50) at the last follow-up visit. In these patients, who had received an average of 1.9 years of miglustat therapy before the observation period, the mean annual progression rate in composite disability score was 0.038 (0.018, 0.059). The annual progression rate in composite disability score appeared highest among patients who had onset of neurological manifestations during infancy or childhood, and lowest in adolescent/adult-onset patients (Fig. 3). While Fig. 3 illustrates a visual trend, no statistical conclusions can be drawn regarding differences in annual progression rates between the age-at-onset subgroups due to the overlap of the 95 % confidence intervals of the mean rates per subgroup.

### Safety observations

Safety information recorded during the observation period is summarized in Table 3; no unexpected safety findings were identified.

**Table 3:** Safety information before miglustat initiation and during NPC Registry follow up

Safety information/event	Time period	Patients, N	Patients with events, n (%)**
Seizures	Pre-treatment: present	90	21 (23)
	During follow-up: worsened*	39	13 (33)
	During follow-up: new*	39	13 (33)
Thrombocytopenia	Pre-treatment: present	90	18 (20)
	During follow-up: present*	80	43^†^ (54)
Tremor	Pre-treatment: present	91	23 (25)
	During follow-up: worsened*	91	6 (7)
	During follow-up: new*	91	2 (2)
Neuropathy	Pre-treatment: present	90	4 (4)
	During follow-up: worsened*	91	7 (8)
	During follow-up: new*	91	1 (1)
Chronic diarrhea^‡^	Pre-treatment: present	91	2 (2)
	During follow-up: present*	92	10 (11)
Other^††^	During follow-up: present*	92	4 (4)

*Event occurred at least once during follow-up; **percentages calculated according to number of patients with available data; ^†^38 mild thrombocytopenia (101–150 × 10^9^/L) and 10 moderate thrombocytopenia (51–100 × 10^9^/L); ^‡^diarrhea lasting >3 months; ^††^other adverse events comprised: dental cavities, loss of consciousness and circulatory collapse, chronic diarrhea and new seizures (all n = 1)

Known safety/tolerability factors associated with miglustat, including chronic diarrhea, thrombocytopenia and seizures, were frequently reported in the overall population. Chronic diarrhea (i.e., diarrhea lasting >3 months) occurred in 11 % of patients during the observation period and in 2 % of patients before treatment. Thrombocytopenia was recorded in a total of 43 (54 %) patients during follow-up and in 18 (20 %) patients before miglustat therapy, and was mild or moderate in almost all (98 %) cases. Seizures were present in a total of 21 (23 %) patients before miglustat therapy, and 13 (33 %) patients with available data had new occurrences of seizures during the observation period. Tremor was present before miglustat initiation in 23 (25 %) patients, and two new occurrences of tremor were reported during follow-up. Neuropathy was present in four (4 %) patients before miglustat initiation and one patient developed a new occurrence of neuropathy during follow-up. ‘Other’ adverse events included one case each of: loss of consciousness and circulatory collapse; chronic diarrhea; new seizures; and dental cavities. In the patient who had dental cavities, miglustat was administered in a sugary beverage. The diarrhea and the seizures were double-reported, once under the actual term and once under ‘other AE’.

## Discussion

Here, we present a longitudinal analysis of functional disability in 92 miglustat-treated patients included from the international NPC Registry. The 92 patients described here received continuous miglustat therapy, with an average observation period of 2 years in the Registry. It should be noted that while the NPC Registry is a disease registry, we only describe findings from miglustat-treated patients here.

Functional disability, evaluated using an established disease-specific scale based on ambulation, manipulation, language, and swallowing [3, 8], remained stable in most NP-C patients during continuous miglustat therapy, and improved in some of them. This is in line with published data from previous studies with miglustat. A retrospective cohort study in 66 NP-C patients demonstrated stable or improved neurological manifestations (using the same composite disability scale employed in the NPC Registry) in over three-quarters of patients during miglustat therapy over a median (range) of 1.46 (0.05–4.51) years [3, 8]. Ambulation and swallowing were also shown to be stable or improved in miglustat-treated adults of a randomized, controlled clinical trial [5]. A pediatric sub-study conducted in parallel with the main randomized trial showed similar findings in children aged 4–12 years [5].

The low number of treatment-naïve patients currently enrolled in the NP-C Registry, and the differences in disease characteristics between these patients and enrolled, treated patients, prevented comparative analyses of miglustat treatment effectiveness. Nevertheless, a degree of comparison can be drawn against natural history data from two published retrospective cohort studies, which assessed disease progression from diagnosis to the start of miglustat therapy. Similar to the Registry, patients in these retrospective cohorts were assessed based on observational methods, from comparable regions and ages at diagnosis [8, 15]. The mean (95 % CI) annual progression rates reported in these two analyses were 0.11 (0.04, 0.18) score units/year in 66 patients over a mean (SD) of 3.1 (3.4) years [8], and 0.12 (0.09, 0.15) score units/year in 57 NP-C patients over a mean (SD) of 5.5 (4.8) years [15]. In the current NPC Registry cohort of miglustat-treated patients, mean (95 % CI) disease progression appears slower (0.038 [0.018, 0.059] score units/year) than that observed among untreated patients in the retrospective cohort studies.

When comparing the annual disease progression of the treated patients from the Registry with the annual disease progression of treated patients in the retrospective cohort [8], it appears that the mean (95 % CI) progression rate in the Registry cohort was greater (0.038 [0.018, 0.059]), than that reported during miglustat treatment in the retrospective cohort (−0.01 [−0.08, 0.06]) [8]. However, the progression rate reported for the retrospective cohort was based on the first 1.5 years of miglustat treatment (i.e., from treatment start), whereas the progression rate calculated for the Registry cohort was based on patients who had already received an average of 1.9 years of miglustat therapy before the observation period. Hence, despite being further along in their treatment course, a large proportion of the Registry patients still showed no progression of neurological disease.

Our analysis of annual disease progression revealed a trend for slower progression in the later neurological onset subgroups and more rapid progression in earlier neurological onset subgroups, which is in line with previous data [1, 8, 15, 16]. Even though disease progression was fastest among the infantile-onset subgroups in this study, stable/improved neurological disease was observed in 33 % (early infantile) to 50 % (late infantile) of treated patients.

Given the progressive, degenerative nature of NP-C, it is difficult to distinguish safety-related, treatment emergent adverse events from those related to disease progression. Nevertheless, based on the safety information reported here we consider that no unexpected safety findings were recorded during the NPC Registry observation period. The most common safety-relevant findings were chronic diarrhea (11 %), thrombocytopenia (54 %) and seizures (33 %). Gastrointestinal disturbances such as mild or moderate diarrhea and flatulence have previously been reported as transient in clinical practice and during clinical trials with miglustat [4, 17, 18].

The high rate of thrombocytopenia among patients in this NPC Registry cohort may be explained by the high proportion of patients in the Registry who had ongoing splenomegaly (39 %): patients with ongoing splenomegaly are more likely to exhibit a mild or moderate degree of thrombocytopenia [3].

Seizures were present in 23 % of NPC Registry patients before miglustat therapy, and during the observation period, new seizures were reported in 33 % of treated patients. It is well known from the literature that seizures are a common feature of the natural history of NPC, and NPC Registry data on seizures that occurred before miglustat therapy (‘pre-miglustat’) support this. Effective seizure control using anti-epileptic agents is considered crucial in optimizing patient quality of life [2, 19].

New occurrences of tremor and neuropathy were relatively infrequent during the observation period. Again, however, it should be borne in mind that 25 % of NPC Registry patients had tremor and 4 % had neuropathy before miglustat initiation.

The clinical profile of miglustat-treated patients from the Registry presented in this paper is consistent with data from previous large cohort studies [1, 11, 12, 20, 21]. The disease characteristics at enrolment of this miglustat-treated group were similar to those reported for the whole NPC Registry cohort as of March 2012, where data from both miglustat-treated and untreated patients were pooled [14]. In addition, the estimated overall participation rate for European countries at the time of data analysis was high (63 %). Given this high rate of patient/physician participation, as well as the wide global coverage of participating centers, the presented miglustat-treated cohort of NPC registry patients can be considered representative of the overall NP-C patient population treated with miglustat, both within the Registry and globally.

This analysis comes with some limitations in terms of recruitment and data interpretation. The fact that the majority of patients with a confirmed diagnosis of NP-C are currently being treated with miglustat has the drawback that there are only a few treatment-naïve patients available for comparison. Moreover, as per health authority commitments, patient recruitment in the NPC Registry is primarily focused on countries where miglustat is approved for the treatment of NP-C. Physicians may not treat patients if they consider miglustat therapy unsuitable for them. For instance, miglustat therapy is not recommended for patients without neurological manifestations [2, 4]. Some patients, in particular pediatric patients, might not be treated with miglustat due to difficulties swallowing a capsule, and some patients might be considered as too severely affected by the disease to benefit from treatment. The Registry untreated cohort therefore might not represent the full spectrum of the disease, and as a consequence untreated Registry patients are considered to be inadequate controls for the treated group. This precludes meaningful comparative analyses of the effectiveness of miglustat therapy.

Another limitation is the retrospective nature of data collection regarding onset of neurological manifestations, which might be subject to recall bias, especially in older patients for whom onset of neurological manifestations may be in the distant past. Despite this potential threat of patient misclassification, the most common disease forms are late-infantile and juvenile- onset NP-C, as previously described [22].

## Conclusion

In summary, this miglustat-treated NP-C cohort is considered representative of the diagnosed and treated NP-C population, displaying typical neurological manifestations across the age-at-neurological onset groups. The majority of patients showed stable or improved neurological manifestations during an average of 2 years of miglustat therapy.
